# SAG/RBX2 is a novel substrate of NEDD4-1 E3 ubiquitin ligase and mediates NEDD4-1 induced chemosensitization

**DOI:** 10.18632/oncotarget.2246

**Published:** 2014-07-23

**Authors:** Weihua Zhou, Jie Xu, Yongchao Zhao, Yi Sun

**Affiliations:** ^1^ Division of Radiation and Cancer Biology, Department of Radiation Oncology, University of Michigan, Ann Arbor, MI; ^2^ Institute of Translational Medicine, Zhejiang University School of Medicine, Hangzhou, Zhejiang, P.R. China

**Keywords:** E3 ubiquitin ligase, NEDD4-1 E3 ligase, Protein ubiquitylation and degradation, SAG E3 ligase

## Abstract

Sensitive to apoptosis gene (SAG), also known as RBX2, ROC2, or RNF7, is a RING component of SCF E3 ubiquitin ligases, which regulates cellular functions through ubiquitylation and degradation of many protein substrates. Although our previous studies showed that SAG is transcriptionally induced by redox, mitogen and hypoxia via AP-1 and HIF-1, it is completely unknown whether and how SAG is ubiquitylated and degraded. Here we report that NEDD4-1, a HECT domain-containing E3 ubiquitin ligase, binds via its HECT domain directly with SAG's C-terminal RING domain and ubiquitylates SAG for proteasome-mediated degradation. Consistently, SAG protein half-life is shortened or extended by NEDD4-1 overexpression or silencing, respectively. We also found that SAG bridges NEDD4-1 via its C-terminus and CUL-5 via its N-terminus to form a NEDD4-1/SAG/CUL-5 tri-complex. Biologically, NEDD4-1 overexpression sensitizes cancer cells to etoposide-induced apoptosis by reducing SAG levels through targeted degradation. Thus, SAG is added to a growing list of NEDD4-1 substrates and mediates its biological function.

## INTRODUCTION

Protein ubiquitylation is carried out by a three-step enzymatic cascade, involving an E1 ubiquitin-activating enzyme, an E2 ubiquitin-conjugating enzyme, and an E3 ubiquitin ligase [1]. HECT (Homologous to E6-AP C-terminus) domain-containing E3s and RING (Really Interesting New Gene) finger-containing E3s are the two major classes of E3s [2]. The HECT catalytic domain forms an E3-ubiquitin thioester intermediate prior to directly transferring the ubiquitin to the bound substrate [3], whereas the RING E3s use a RING finger domain to recruit ubiquitin-loaded E2 and transfer the ubiquitin from E2 to a lysine residue on a targeted substrate via an isopeptide bond [4]. The multi-unit Cullin RING ligase (CRL) is the largest family of E3 ubiquitin ligases with its RING component being either RBX1 or RBX2 [5, 6]. Both family members contain an evolutionarily conserved RING finger domain at the C-terminus, which is required for the ubiquitin ligase activity of CRLs [7, 8].

RBX2, also known as SAG, ROC2, or RNF7, was first cloned in our laboratory as a redox-inducible protein [5]. Our subsequent *in vitro* and *in vivo* studies showed that SAG is an anti-apoptotic cellular survival protein whose overexpression protects cells from apoptosis induced by various stimuli [9, 10], whereas its silencing by siRNA or deletion by gene knockout induces apoptosis [11, 12]. SAG promotes the stage-dependent degradation of c-Jun and IκBα, thus regulating skin carcinogenesis induced by DMBA-TPA [9]. Our most recent results showed that SAG is an onco-cooperating gene, required for lung tumorigenesis induced by a mutant Kras [13]. Although our previous studies showed that SAG is subjected to the regulation of AP-1 [14] and HIF-1 [15] at the transcriptional level, it is unknown how SAG is regulated at the post-translational level and by which E3 ligase.

Human NEDD4-1 is a HECT domain-containing E3 ubiquitin ligase. The NEDD4 family consists of nine members with NEDD4-1 (NEDD4) and NEDD4-2 (NEDD4L) being most closely related to each other [2]. Despite ubiquitous expression of NEDD4-1 and NEDD4-2, it appears that the two proteins have distinct functions by targeting specific proteins for ubiquitylation [for review see ref. [16]]. Early studies showed that NEDD4-1 promotes PTEN polyubiquitylation for targeted degradation [17], and PTEN monoubiquitylation for targeted nucleus localization [18]. However, the recent mouse *in vivo* studies showed that *Nedd4-1* knockout neither changes the PTEN level, nor PTEN subcellular localization [19, 20], raising the issue of whether PTEN is a physiological substrate of NEDD4-1. Besides controversial PTEN, other reported substrates of NEDD4-1 include growth factor receptors, such as IGF1R [21, 22] and FGFR1 [23], AKT [24], the RING-finger E3, Cbl [25], along with a few others [for review see ref. [26]].

A recent large scale proteomic study identified SAG as a putative NEDD4-1 binding partner [27]. In this study, we followed-up this lead and reported here that SAG indeed binds to NEDD4-1 and is subjected to NEDD4-1 mediated ubiquitylation and degradation. Our study demonstrates that SAG is a novel substrate of NEDD4-1.

## RESULTS

### The protein levels of SAG and NEDD4-1 are inversely correlated and SAG binds to NEDD4-1 via its RING domain

**Figure 1:**
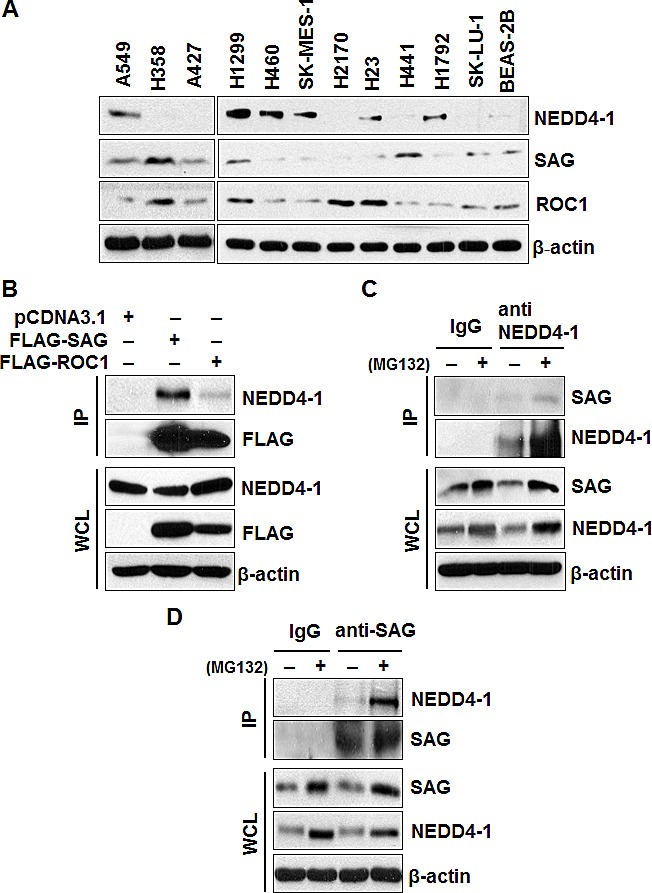
Levels of SAG and NEDD4-1 in lung cancer cells and SAG-NEDD4-1 binding (A). Protein levels of NEDD4-1, SAG, and ROC1 in multiple lung cancer cell lines and a normal cell line, BEAS-2B. Cell lysates were prepared from indicated cell lines, followed by Western blotting with equal amount protein loaded, using indicated antibodies. (B). SAG immunoprecipitates endogenous NEDD4-1. 293 cells were transiently transfected with FLAG-SAG and FLAG-ROC1, along with pCDNA3.1 vector control. Cells were lysed and immunoprecipitated with FLAG antibody, followed by IB with indicated antibodies. (C&D). Binding of endogenous SAG and NEDD4-1. H1299 cells were left untreated or treated with 10 μM MG132 for 4 hrs prior to harvesting. Whole cell extracts were immunoprecipitated with antibodies against NEDD4-1 (C) or SAG (D), along with IgG control, followed by Western blotting with indicated antibodies.

In a recent large scale proteomic study, SAG was identified as a binding partner of human NEDD4-1, but not NEDD4-2 [27]. Since it is unknown how SAG is ubiquitylated and degraded, and by which E3 ligase, we hypothesized that NEDD4-1 may be an E3 ligase for SAG. We first determined potential correlation in the protein levels between SAG and NEDD4-1 in multiple human lung cancer cell lines, and found that the level of SAG, but not its family member RBX1, is largely correlated in a inverse manner with NEDD4-1 (Figure 1A). We next determined whether the two proteins would bind to each other by an IP pull-down assay. Indeed, ectopically expressed SAG, but to a lesser extent, ROC1, pulled down endogenous NEDD4-1 in 293 cells (Figure 1B). We further determined endogenous binding of two proteins and found that SAG was detected in NEDD4-1 immunoprecipitates, but not in the IgG control. Reciprocally, NEDD4-1 was detected in SAG immunoprecipitates (Figure 1C&D). Moreover, SAG-NEDD4-1 binding was significantly enhanced when protein degradation was inhibited in the presence of MG132 (Figure 1C&D). Thus, SAG binds to NEDD4-1 under the physiological conditions.

**Figure 2:**
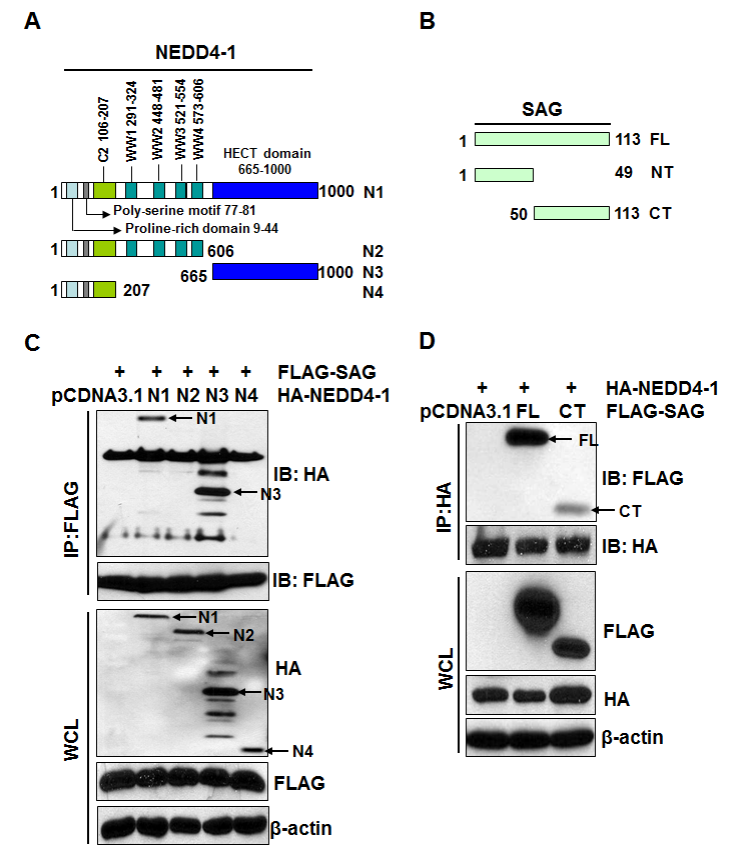
NEDD4-1 interacts with SAG-CT via its HECT domain (**A&B**). Schematic representation of NEDD4-1 and SAG domain structures. (**C**). NEDD4-1 interacts with SAG through its HECT domain. 293 cells were transiently transfected with N1, N2, N3, N4 and pCDNA3.1 vector control, respectively, in combination with FLAG-tagged full-length SAG. Cells were lysed and immunoprecipitated with FLAG antibody, followed by IB with indicated Abs. (**D**). SAG interacts with NEDD4-1 through its C-terminal RING domain. 293 cells were transiently transfected with FL, CT, and pCDNA3.1 vector control, respectively, in combination with HA-tagged full-length NEDD4-1. Cells were lysed and immunoprecipitated with HA antibody, followed by IB with indicated Abs.

The NEDD4 family contains an N-terminal C2 domain for membrane targeting, a central region containing WW domains for protein–protein interaction, and a C-terminal catalytic HECT domain for ubiquitin protein ligation [28] (Figure 2A). Substrate recruitment by NEDD4 proteins is mediated via WW domains that recognize the PY (LPxY or PPxY) motifs in target proteins [29]. Although the majority of the top substrates of NEDD4-1 harbored the expected PY motifs, several potential substrates bound NEDD4-1 through an unknown mechanism [27]. Given that SAG does not contain such a PY motif, we defined the binding domains on both proteins that mediate their binding. A series of HA-tagged NEDD4-1 deletion mutants were constructed (Figure 2A), along with the FLAG-tagged SAG N-terminal (NT) fragment and the C-terminal (CT) RING domain (Figure 2B). The two-way pull down assay revealed that full length HA-NEDD4-1 and its deletion mutant HA-NEDD4-1-N3 were detected in FLAG-SAG immunoprecipitates by anti-FLAG antibody (Figure 2C), whereas SAG was detected in immunoprecipitates of wt and HA-NEDD4-1-N3 mutant by anti-HA antibody (Figure S1A), indicating that NEDD4-1 interacts with SAG via its HECT domain. Reciprocally, both wt FLAG-SAG and FLAG-SAG RING domain were detected in HA-NEDD4-1 immunoprecipitates by anti-HA antibody (Figure 2D). Likewise, HA-NEDD4-1 was detected in immunoprecipitates of wt FLAG-SAG and FLAG-SAG RING domain by anti-FLAG antibody (Figure S1B). Note that expression of the SAG N-terminal fragment was undetectable (data not shown). Taken together, our results indicate that SAG-NEDD4-1 binding is mediated by the HECT domain on NEDD4-1 and the RING domain on SAG. Interestingly, both domains are required for the ligase activity of either protein.

### NEDD4-1/SAG/CUL-5 forms a tri-complex

**Figure 3:**
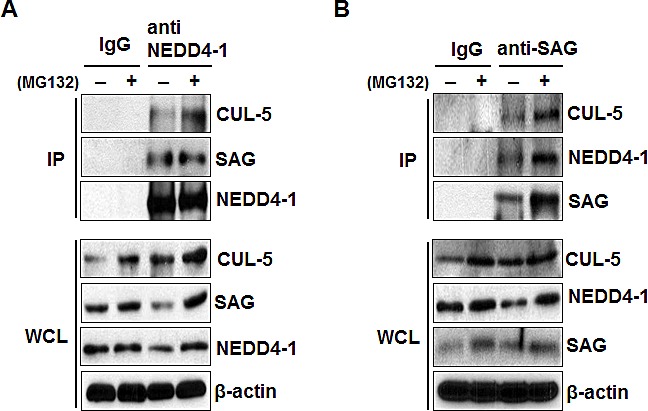
SAG bridges NEDD4-1 and CUL-5 to form a NEDD4-1/SAG/CUL-5 tri-complex (**A&B**). NEDD4-1, SAG, and CUL-5 bind to each other under physiological condition. H1299 cells were left untreated or treated with 10 μM MG132 for 4 hrs prior to harvesting. Whole cell extracts were immunoprecipitated with antibodies against NEDD4-1 (**A**) or SAG (**B**), along with IgG control, followed by Western blotting with indicated antibodies.

It has been previously shown that a) under physiological conditions, SAG preferentially binds with CUL-5 [30], and b) SAG, through its N-terminal domain (NTD) binds to the C-terminal domain (CTD) of CUL-5 [31, 32]. We, therefore, tested our hypothesis that NEDD4-1/SAG/CUL-5 might form a tri-complex under physiological conditions by a pull-down assay. Indeed, both SAG and CUL-5 were detected in NEDD4-1 immunoprecipitates (Figure 3A). Likewise, both NEDD4-1 and CUL-5 were detected in SAG immunoprecipitates (Figure 3B). Furthermore, treatment with proteasome inhibitor MG132 increased the SAG levels as well as the binding among the three components (Figure 3A&B). Taken together, our results indicated that SAG binds to CUL-5 via its NTD [31, 32] and NEDD4-1 via its CTD to form a NEDD4-1/SAG/CUL-5 tri-complex under physiological conditions.

### NEDD4-1 regulates SAG protein level by promoting its poly-ubiquitylation

**Figure 4:**
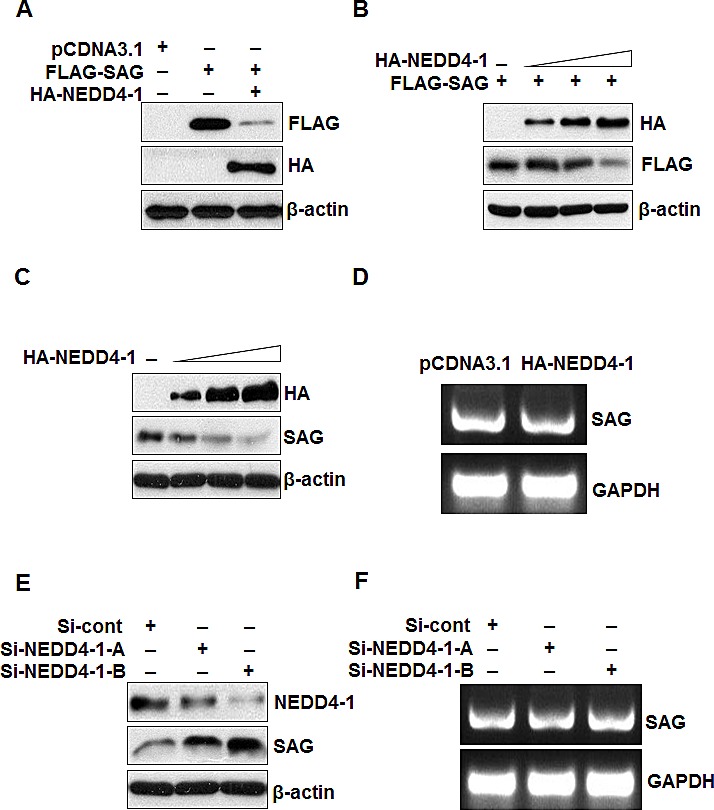
NEDD4-1 negatively regulates SAG protein level (**A&B**). NEDD4-1 negatively regulates SAG protein level. 293 cells were transfected with pCDNA3.1 vector, FLAG-SAG, or FLAG-SAG in combination with HA-NEDD4-1 (**A**), or FLAG-SAG in combination with increasing amounts of HA-NEDD4-1 (**B**); Whole cell extracts were analyzed by Western blotting with FLAG or HA antibodies. (**C**) NEDD4-1 over-expression decreased the endogenous SAG protein level. H358 cells were transfected with increasing amounts of HA-NEDD4-1. Whole cell extracts were analyzed by Western blotting with HA or SAG antibodies. (**D**). NEDD4-1 ectopic expression has no effect on SAG mRNA level. H358 cells were transfected with HA-NEDD4-1. 48 hrs post-transfection, total RNA was isolated and SAG mRNA was determined by RT-PCR. (**E**). NEDD4-1 depletion increased SAG protein level. SK-MES-1 cells were transfected with two independent siRNA targeting NEDD4-1, along with control RNAi. Whole cell extracts were analyzed by Western blotting with antibodies against NEDD4-1 and SAG. (**F**). NEDD4-1 depletion has no effect on SAG mRNA level. SK-MES-1 cells were transfected with two independent siRNA targeting NEDD4-1, along with control RNAi. 48 hrs later, total RNA was extracted for RT-PCR assay.

Having detected inversely correlated protein levels and a physical interaction between NEDD4-1 and SAG, we next determined whether SAG protein level is regulated by NEDD4-1. In a co-transfection experiment in 293 cells, when NEDD4-1 was co-transfected, the level of ectopically expressed SAG was reduced in a dose dependent manner (Figure 4A&B). We further transfected NEDD4-1 into H358 lung cancer cells, which show a low level of NEDD4-1 and a high level of SAG (Figure 1A), and found a dose dependent reduction of endogenous SAG (Figure 4C). Furthermore, ectopic expression of HA-NEDD4-1 had no effect on SAG mRNA level (Figure 4D). Consistently, we performed a siRNA-based knockdown experiment in SK-MES-1 lung cancer cells, where NEDD4-1 levels are high, but SAG levels are moderate, and found that the endogenous SAG level increased when NEDD4-1 was knocked down by two independent siRNAs in a manner dependent on the level of NEDD4-1 knockdown (Figure 4E). Again, NEDD4-1 knockdown didn't change the level of SAG mRNA (Figure 4F). These data collectively indicate that NEDD4-1 negatively regulates the SAG protein levels.

**Figure 5:**
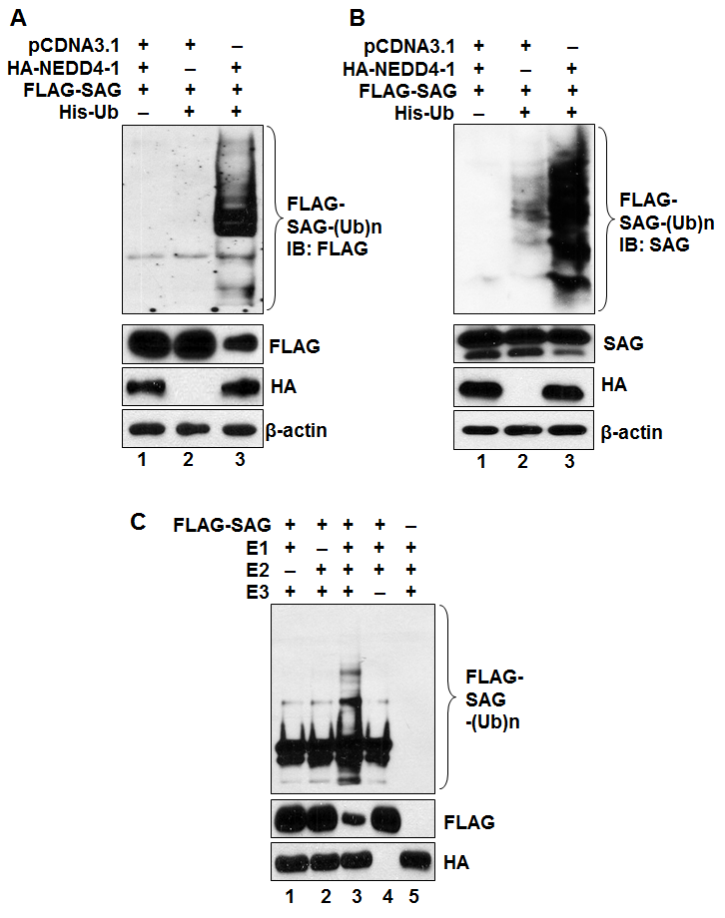
NEDD4-1 facilitates poly-ubiquitylation of SAG (**A&B**). NEDD4-1 promotes poly-ubiquitylation of SAG *in vivo*. 293 cells were cotransfected with indicated vectors encoding HA-NEDD4-1 or/and FLAG-SAG in combination with His-ubiquitin. Whole cell extracts and Ni-NTA affinity-purified fractions were analyzed by Western blotting with anti-FLAG (**A**) or anti-SAG (**B**) antibodies. (**C**). NEDD4-1 promotes ubiquitylation of SAG *in vitro*. HA-NEDD4-1 E3 was precipitated from 293 cells after being transiently transfected with HA-NEDD4-1 and eluted with 3xHA peptide. FLAG-tagged SAG was pulled down by FLAG beads after transfection into 293 cells. HA-NEDD4-1 E3 and FLAG-tagged SAG were added into a reaction mixture containing ATP, ubiquitin, E1 and E2, followed by constant mixing for 1hr. The reaction mixture was then loaded onto PAGE gel for IB using anti-FLAG Ab.

To elucidate the molecular mechanism underlying NEDD4-1-mediated negative regulation of SAG protein level, we first determined whether SAG is subjected to poly-ubiquitylation by ectopically expressed NEDD4-1. A cell-based ubiquitylation assay was performed where ubiquitylated proteins were captured with nickel-nitrilotriacetic acid (Ni-NTA) affinity chromatography from 293 cells transfected with pCDNA3.1 vector, FLAG-SAG, His-ubiquitin and HA-NEDD4-1, followed by detection of SAG ubiquitylation by anti-FLAG antibody (for exogenous SAG) (Figure 5A) or anti-SAG antibody (for both exogenous and endogenous SAG) (Figure 5B). In both cases, NEDD4-1 promoted substantial poly-ubiquitylation of SAG (Figure 5A&B), indicating that NEDD4-1 promotes the ubiquitylation of both exogenous and endogenous SAG.

We further determined SAG poly-ubiquitylation, using an *in vitro* purified system containing E1, E2, and E3 (HA-NEDD4-1), and found that HA-NEDD4-1 induced SAG ubiquitylation in a manner dependent on both E1 and E2 (Figure 5C; lane 3). NEDD4-1-mediated poly-ubiqutination of SAG also leads to a significant reduction of SAG protein, as measured by a direct Western blotting analysis (Figure 5C; lane 3). Taken together, our data indicate that NEDD4-1 mediates SAG poly-ubiquitylation and subsequent degradation.

### SAG protein half-life is shortened by NEDD4-1 overexpression and extended by NEDD4-1 knockdown

**Figure 6:**
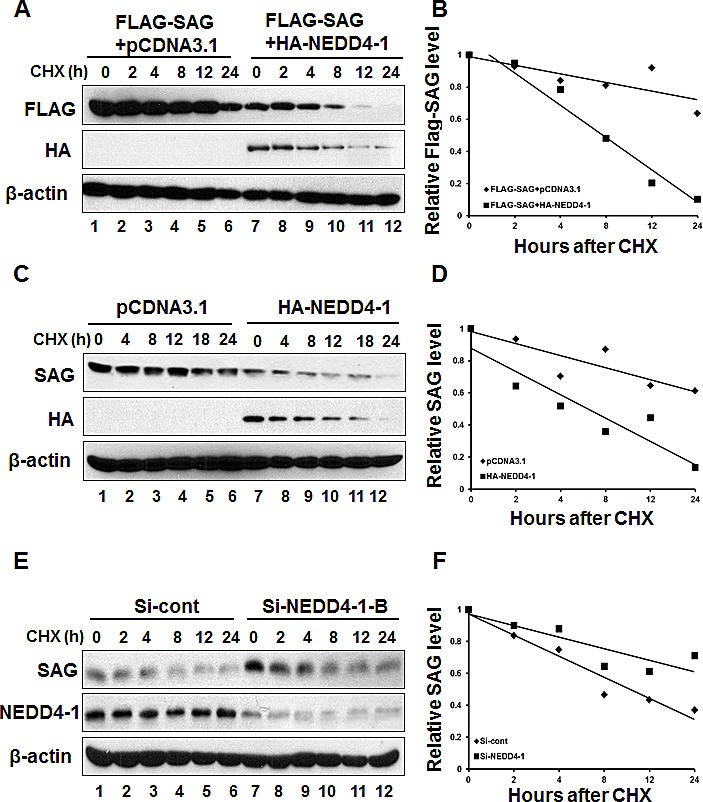
NEDD4-1 shortens the half-life of SAG protein (**A&B**). NEDD4-1 shortened the half-life of exogenous SAG protein. After transfection with relevant plasmids for 48 hrs, cells were switched to fresh medium (10% FBS) containing cycloheximide (CHX) and incubated for indicated time periods before being harvested for Western blotting (**A**). The band density was quantified using ImageJ software and plotted (**B**). (**C&D**). NEDD4-1 shortened the half-life of endogenous SAG protein. HA-NEDD4-1 was transfected into H358 cells for 48 hrs. Cells were then cultured in fresh medium (10% FBS) containing CHX and incubated for indicated time periods prior to being harvested for Western blotting (**C**). The band density was quantified using ImageJ software and plotted (**D**). (**E&F**). NEDD4-1 RNAi silencing extended protein half-life of endogenous SAG. SK-MES-1 cells were transfected with either control RNAi, or Si-NEDD4-1-B for 48 hrs. Cells were cultured in fresh medium containing CHX and incubated for indicated time periods before being harvested for Western blotting (**E**). The band density was quantified using ImageJ software and plotted (**F**).

We continued to determine whether NEDD4-1 shortened SAG protein half-life. In a cotransfection experiment, HA-NEDD4-1 overexpression decreased the basal level of exogenous SAG and markedly shortened its protein half-life from more than 24 hrs to ~ 8 hrs (Figure 6A&B, lanes 7-12). Likewise, the basal level of endogenous SAG was also reduced significantly with its protein half-life shortened from longer than 24 hrs to ~8 hrs, after HA-NEDD4-1 transfection into SAG high but NEDD4-1 low expressing H358 cells (Figure 6C&D, lanes 7-12). Consistently, siRNA knockdown of NEDD4-1 caused the accumulation of SAG basal level and extended the protein half-life of endogenous SAG from 8 hrs to more than 24 hrs in SK-MES-1 cells, which express a high level of NEDD4-1 (Figure 6E&F, lanes 7-12). Taken together, our results support the notion that SAG is a new substrate of NEDD4-1 E3 ligase, which ubiquitylates it for targeted degradation.

### Overexpression of HA-NEDD4-1 sensitizes lung cancer cells to etoposide-induced apoptosis

**Figure 7:**
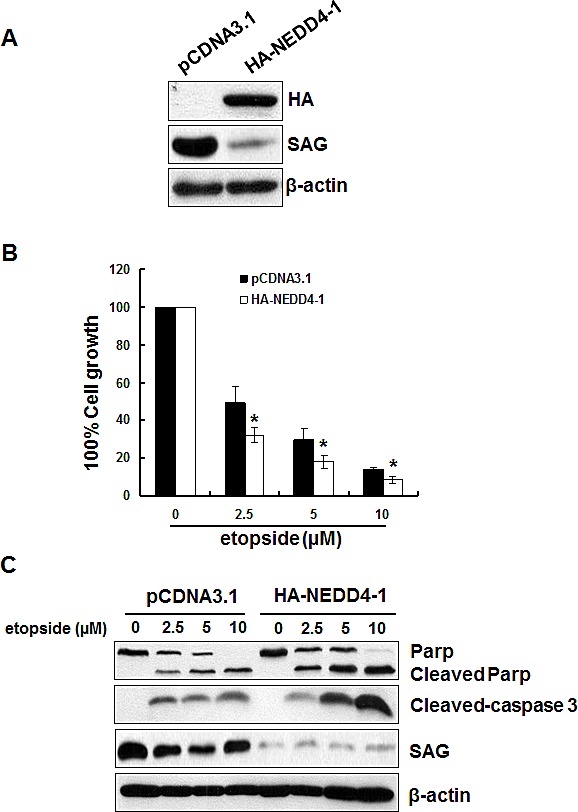
Overexpression of HA-NEDD4-1 sensitizes lung cancer cells to etoposide-induced apoptosis (**A**). Stable H358 clones, expressing HA-NEDD4-1 or pCDNA3.1 vector control cells were subjected to Western blotting using indicated antibodies. (**B&C**). Overexpression of HA-NEDD4-1 sensitizes lung cancer cells to etoposide-induced apoptosis. H358 cells expressing HA-NEDD4-1 or pCDNA3.1 were seeded into 96-well or 60mm dishes, respectively. Cells were then incubated in the fresh medium containing various doses of etoposide for 48 hrs before being analyzed by ATPlite assay (**B**) or being harvested for Western blotting (**C**).

SAG has been shown to promote cell proliferation and protect cancer cells from apoptosis induced by various stimuli [for review see ref. [33]]. We therefore determined whether NEDD4-1-mediated SAG degradation would inhibit cancer cell growth and promote apoptosis. To this end, we established H358 stable cell line overexpressing HA-NEDD4-1 with corresponding reduction of SAG (Figure 7A). ATPlite-based proliferation assay showed that ectopic NEDD4-1 expression significantly sensitized H358 cells to etoposide-induced growth suppression in all three concentrations tested (Figure 7B). The nature of growth suppression was found to be mediated by apoptosis, as evidenced by increased levels of cleaved PARP and caspase-3 (Figure 7C). Taken together, these results indicated that by reducing endogenous SAG level, NEDD4-1 overexpression sensitized lung cancer cells to etoposide-induced growth suppression by enhancing apoptosis.

## DISCUSSION

Our previous studies have shown that SAG is positively regulated by transcription factors AP-1 [14] and HIF-1 [15] at the transcriptional levels. Herein, we demonstrated that SAG is negatively regulated by NEDD4-1 E3 ubiquitin ligase at the post-translational level. The following lines of evidence support the notion that SAG is a novel substrate of NEDD4-1. First, SAG and NEDD4-1 bind together under the physiological conditions via the RING and HECT domains, respectively. This is an atypical and a non-canonical manner, since NEDD4-1 contains several WW domains, which are the protein interaction modules for the binding of NEDD4-1 to its target proteins containing the PY (LPxY or PPxY) motifs [29]. SAG neither contains PY motif, nor binds to the WW domains of NEDD4-1. Nevertheless, the HECT domain of NEDD4-1 has also been shown to interact with the N-terminal region of PTEN and mediate its ubiquitylation [34]. A similar substrate recognition mechanism is employed by Rsp5, the only ortholog of NEDD4-1 in yeast [35]. Second, the protein levels of SAG in multiple human lung cancer cell lines are inversely correlated with the levels of NEDD4-1. NEDD4-1 overexpression reduces SAG levels in a dose dependent manner, whereas NEDD4-1 knockdown via siRNA causes SAG accumulation, also in a dose dependent manner. Third, NEDD4-1 promotes SAG poly-ubiquitylation and degradation, as demonstrated by both *in vitro* and *in vivo* ubiquitylation assays. Finally, the SAG protein half-life is shortened by NEDD4-1 overexpression, but extended by its knockdown. Thus, SAG is added to a growing list of NEDD4-1 substrates.

Another novel observation made in this study is that SAG binds to CUL-5 probably via its NTD [31, 32] and NEDD4-1 via its CTD, thus bridging NEDD4-1 and CUL-5 to form a NEDD4-1/SAG/CUL-5 tri-complex. It was reported that the C-terminal RING domain of RBX1/ROC1 mediates the binding of RBX1/ROC1 with ubiquitin-loaded E2 conjugation enzyme [8]. Although not determined, it is very likely that is the case for SAG, since RBX1 and SAG two family members shared the same ligase activity, as assayed by the *in vitro* ubiquitylation assay [12, 36]. Thus, NEDD4-1 binding of SAG not only promotes SAG ubiquitylation and degradation, but also prevents its binding with ubiquitin-loaded E2, leading to abrogation of SAG ligase activity. It was also reported that SAG family member RBX1/ROC1 has capacity to undergo self-ubiquitylation and degradation, whereas its association with cullins stabilize it by preventing degradation [37]. Again, although not determined, it is most likely that SAG would also undergo auto-ubiquitylation, which would be prevented when it forms a complex with cullins, particularly CUL-5. Given the fact that NEDD4-1 promotes SAG ubiquitylation and degradation even when SAG forms a stable complex with CUL-5 (Figures 3&5), we conclude that SAG degradation via NEDD4-1-mediated ubiquitylation, not by self-ubiquitylation, contributes to chemosensitization (Figure 7).

As a redox-inducible antioxidant protein, SAG protects cells from apoptosis induced by various stimuli [5], in a RING domain dependent manner. Importantly, recent studies have begun to reveal that SAG might function as an oncoprotein that is frequently overexpressed in human carcinomas of lung, colon, stomach and liver (for review see ref. [33]) and required for lung tumorigenesis, triggered by a mutant Kras [13]. Consistent with our previous observation that SAG silencing sensitized cancer cells to etoposide-induced cell killing [38], NEDD4-1 overexpression, which reduces SAG level, also sensitized lung cancer cells to etoposide-induced growth suppression via apoptosis induction (Figure 7). Hence, inactivation of SAG either by siRNA silencing of itself [11, 38], or by overexpression of its upstream E3 ligase, NEDD4-1 (this study), or by its small molecule inhibitor, MLN4924 [39], all induces apoptosis, further supporting the notion that SAG E3 ligase is an attractive anti-cancer target.

## METHODS

### Plasmids, siRNA, and transfection

HA-NEDD4-1 plasmid was obtained from Dr. Xuejun Jiang, Memorial Sloan-Kettering Cancer Center [17]. FLAG-SAG plasmid was previously constructed in our lab [5]. The NEDD4-1 deletion mutants (N2: 1–606; N3: 605–1000; N4: 1–207) and SAG deletion mutants (NT: 1-49; CT: 50-113) were subcloned into HA-tagged or FLAG-tagged pCDNA3.1 plasmids, respectively. The sequences of two siRNAs targeting NEDD4-1 were selected based upon published literature: NEDD4-1 RNAi-A, UAGAGCCUGGCUGGGUUGUUU [40], and NEDD4-1 RNAi-B, UUCCAUGAAUCUAGAAGAACA [41]. The sequence for scrambled control siRNA is AUUGUAUGCGAUCGCAGACUU. Transfection of the plasmids and siRNAs were carried out using Lipofectamine 2000 (Invitrogen).

### Co-immunoprecipitation and immunoblotting

To immunoprecipitate endogenous proteins, whole cell extracts were pre-cleared with normal IgG-AC (Santa Cruz) followed by overnight incubation at 4°C with antibody against NEDD4-1 (Santa Cruz; #sc-25508) and SAG [11]. To immunoprecipitate exogenously expressed FLAG-tagged or HA-tagged proteins, the pre-cleared cell lysates were incubated with FLAG beads (Sigma) or with HA antibody (Roche) for 3 hrs followed by incubation with protein A&G beads (Santa Cruz) at 4°C overnight with rotation. The beads were washed three times with lysis buffer, and the immunoprecipitation complexes were subjected to SDS-PAGE.

Whole-cell lysates were prepared and subjected to immunoblotting analysis using antibodies against SAG [11], ROC1 [42], HA (Roche), FLAG (Sigma), CUL-5 (Santa Cruz), NEDD4-1 (Cell Signaling; #3607), Parp (Cell signal), Cleaved-caspase-3 (Cell Signal), and β-actin (Sigma)

### The *in vivo* ubiquitylation

The 293 cells were cotransfected with FLAG-SAG, HA-NEDD4-1 and His-HA-ubiquitin, along with pCDNA3.1 vector control. *In vivo* ubiquitylation assays were performed as previously described using Ni-beads pull-down [14].

### The *in vitro* ubiquitylation

HA-NEDD4-1 was precipitated from HA-NEDD4-1 transfected 293 cells and eluted with 3xHA peptide (Sigma), serving as the source of E3. FLAG-tagged SAG was pulled down by FLAG bead (Sigma) after transfection into 293 cells, serving as substrate. The reaction was carried out at 30 °C for 1 hr in 30 μL reaction buffer (40 mM Tris-HCl, pH 7.5, 2 mM DTT, 5 mM MgCl_2_) in the presence of SAG (bound to FLAG beads), ubiquitin (Boston Biochem), E1 (Boston Biochem), recombinant UbcH5c/E2 (Boston Biochem), ATP, and HA-purified NEDD4-1 E3. After the reaction, the beads were washed three times with cell lysis buffer to remove non-SAG-conjugated ubiquitin. The washed beads were then resuspended in 25 μL 2xSDS-PAGE sample buffer for SDS-PAGE and detected by IB with antibodies against FLAG-tag or SAG.

### ATPlite cell proliferation assay

Stable H358 cells expressing HA-NEDD4-1 were established by transfection with HA-NEDD4-1, followed by G418 selection for 2 weeks. The stable clones were pooled and seeded into 96-well plates with 2,000 cells per well in triplicate, along with the Vector control cells. Next day, cells were treated with DMSO control or etoposide (2.5, 5, 10 μM) for 48 hrs, and then subjected to ATPlite cell proliferation assay (Perkin-Elmer).

## SUPPLEMENTARY MATERIAL FIGURE


