# Supplementary material for: SAG/RBX2 is a novel substrate of NEDD4-1 E3 ubiquitin ligase and mediates NEDD4-1 induced chemosensitization
Source: Oncotarget. 2014 Jul 23;5(16):6746–55. doi: 10.18632/oncotarget.2246 (PMC4196160; doi:10.18632/oncotarget.2246)
Supplement: Supplementary file 1 [file oncotarget-05-6746-s001.pdf]

SAG/RBX2 is a novel substrate of NEDD4-1 E3 ubiquitin ligase and mediates NEDD4-1 induced chemosensitization

Supplementary Material

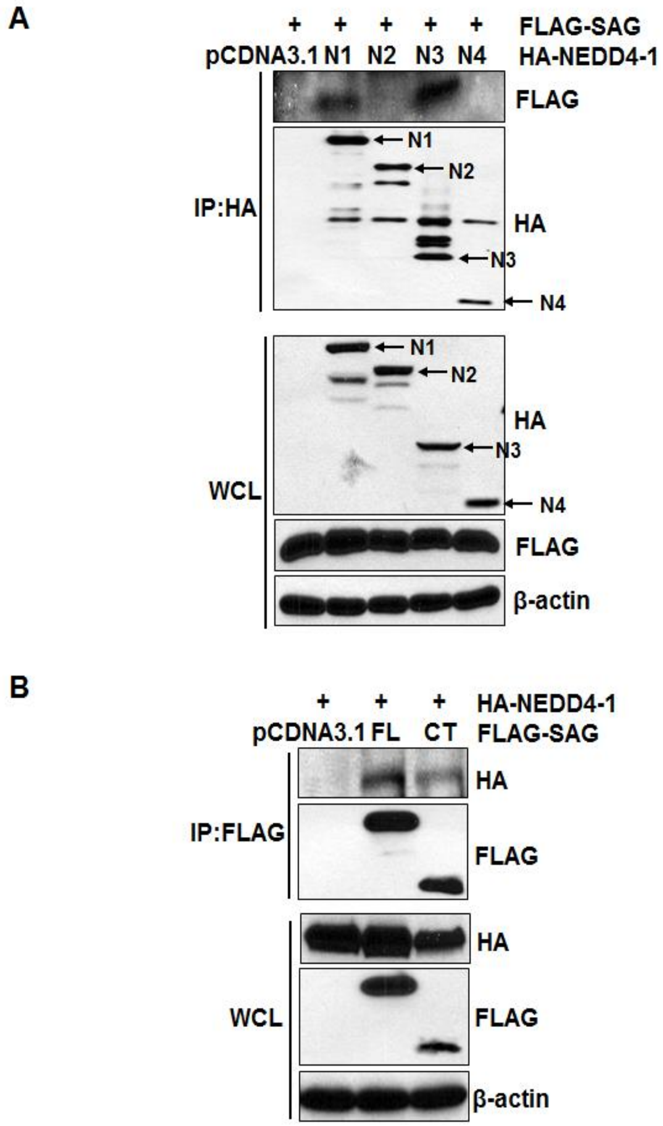

Figure S1: NEDD4-1 interacts with SAG-CT via its HECT domain. (A). NEDD4-1 interacts with SAG through its HECT domain. 293 cells were transiently transfected with indicated plasmids in combination with FLAG-tagged full-lenth SAG. Cells were lysed and immunoprecipitated with HA antibody, followed by IB with indicated antibodies. **(B)** SAG interacts with NEDD4-1 through its C-terminus. 293 cells were transiently transfected with indicated plasmids, in combination with HA-tagged full-lenth NEDD4-1. Cells were lysed and immunoprecipitated with FLAG antibody, followed by IB with indicated antibodies.
